# Celiac plexus neurolysis for abdominal cancers: going beyond pancreatic cancer pain

**DOI:** 10.1097/PR9.0000000000000930

**Published:** 2021-05-12

**Authors:** Vats T. Ambai, Vinita Singh, David W. Boorman, Nathan J. Neufeld

**Affiliations:** a Department of Graduate Medical Education - Transitional Year Residency Program, Northside Hospital - Gwinnett, Lawrenceville, GA, USA; b Department of Anesthesiology, Emory University School of Medicine, Atlanta, GA, USA; c Department of Pain and Palliative Care, Cancer Treatment Centers of America, Newnan, GA, USA

**Keywords:** Celiac plexus neurolysis, Cancer pain, Pancreatic cancer, Gastrointestinal cancer, Hepatobiliary cancer, Quality of life

## Abstract

**Introduction:**

Celiac plexus neurolysis (CPN) has been verified for mitigating pancreatic cancer pain. However, information regarding CPN's use beyond this remains limited.

**Objectives:**

Identify which cancers benefit from CPN, which symptoms improve, and when symptoms improve.

**Methods:**

Retrospective analysis was conducted on 173 patients who received CPN for pain caused by various malignancies. Mean symptom changes on the MD Anderson Symptom Inventory (MDASI) from baseline to 2 weeks, 1 month, and 2 months after CPN were analyzed overall and then by cancer type: pancreatic (all stages and stages III–IV), hepatobiliary, and nonpancreatic, nonhepatobiliary gastrointestinal (NPNH-gastrointestinal).

**Results:**

Eighty-two pancreatic, 43 NPNH-gastrointestinal, 14 hepatobiliary, and 34 patients with other cancers met inclusion criteria. Statistically significant changes included decrease in the pain score at 1 month by 1.01 points for all cancers, 1.65 points for all pancreatic cancers, and 1.88 points for late-stage pancreatic cancers. At 2 months, pain decreased by 1.50 points for all cancers, 1.68 points for all pancreatic cancers, 2.37 points for late-stage pancreatic cancers, and 1.50 points in NPNH-gastrointestinal cancers. At 2 months, quality of life improved by 1.07 points for all cancers and 1.53 points for all pancreatic cancers. Sleep improved at 2 months for all cancers by 0.73 points and 1.60 points in late-stage pancreatic cancers. At 2 months, pancreatic cancer patients improved in general activity by 0.93 points, walking by 1.00 points, and working by 1.12 points.

**Conclusion:**

Celiac plexus neurolysis can decrease cancer symptom burden beyond pain including quality of life and sleep for pancreatic and nonpancreatic cancers, as well as general activity for pancreatic cancers.

## Abstract 2

Celiac plexus neurolysis is primarily used for pancreatic cancer pain, but other symptoms and other cancers may also benefit from this intervention.

## 1. Introduction

Newly diagnosed cancer cases are expected to reach 1.8 million in the United States each year, with more than 15% of these cancers being visceral cancers that send their pain signals through the celiac plexus.^19^ Because these patients grapple with their diagnoses, they will experience a variety of unpleasant symptoms, with pain being one of the most pervasive. Their sensation of pain may originate from any combination of nociceptive (visceral or somatic), neuropathic, and psychogenic factors, ultimately being treated using a laddered approach beginning with nonsteroidal anti-inflammatory drugs then progressing to opioids.^4^ For epigastric cancer patients who cannot tolerate the side effects of opioids or those who continue to suffer from pain despite opioid use, the World Health Organization advises supplementing treatment with percutaneous celiac plexus neurolysis (CPN).^23^

Celiac plexus neurolysis has been verified for treating pancreatic cancer pain, but the broad anatomic reach of the celiac plexus makes it likely that other symptoms and cancers may also benefit. In previous studies of pancreatic cancer patients, the physical, emotional, and functional changes resulting from CPN amount to improved outcomes.^18,20^ Although these studies were conducted in limited numbers of patients, and the timeframe and extent of symptom changes varied among the studies, enough evidence has been presented to state that neurolytic destruction of the celiac plexus is a safe and tolerable procedure when compared with opioid-exclusive therapies.^2,10^ As for CPN's use in other cancers, few studies have ventured beyond pancreatic cancer. The handful that have broached the subject are equally limited for sample size but have shown results that prompt additional research.^5–7,10,11,15^

This retrospective study analyzed data from 173 patients at a single hospital to identify which cancers benefit, which symptoms improve, and which stages are best to provide the procedure to patients suffering from unresectable abdominal malignancies.

## 2. Methods

**Figure 1.**
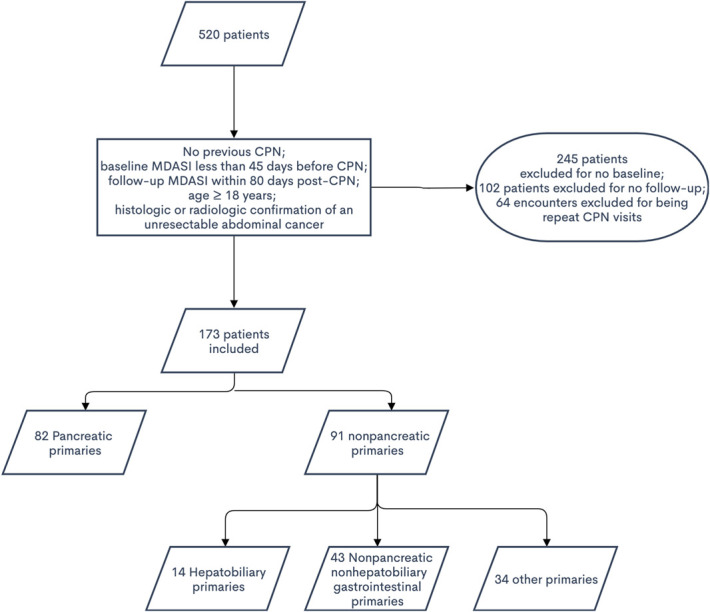
Patient selection.

This study was approved by the Western Institutional Review Board in accordance with the policies of the Cancer Treatment Centers of America—Atlanta. A waiver was granted for informed consent, and patient information was deidentified to retain anonymity. At the time of data collection, records available for retrospective analysis spanned 46 months and included 520 encounters. Inclusion criteria used to identify eligible patients were: age ≥18 years, histologic or radiologic confirmation of an unresectable abdominal cancer, completion of the CPN procedure, availability of a baseline MD Anderson Symptom Inventory (MDASI) survey ≤45 days before CPN, and at least one follow-up MDASI survey ≤80 days after CPN. A total of 173 patients fulfilled requirements for inclusion in the study (Fig. 1). Some patients had a repeat CPN performed after their initial CPN, in which case surveys before their second CPN were included and any surveys collected after the second CPN were excluded. Data points included the date of cancer diagnosis, date of first pain consultation, date of CPN procedure, date of registered death, dates of any subsequent CPN procedures, historical diagnoses, and responses to the MDASI. A single author aggregated the data and reviewed it to ensure consistency.

All patients in the study obtained a pain management consultation for abdominal pain originating in the region supplied by the celiac plexus in the setting of imaging-confirmed unresectable cancer with opioid intolerance or opioid treatment failure. Celiac plexus neurolysis was performed by the same physician, at a single facility under monitored anesthesia care with the use of fluoroscopic guidance to achieve a bilateral retrocrural approach. Patients were placed in the prone position, a sterile field was prepared, and an oblique view was obtained to superimpose the transverse spinous process of L1 over the L1 vertebral body. Five mL of 1% lidocaine was then used as a local anesthetic. This was followed by insertion of a 22-gauge 5-inch needle, which was advanced to the lateral edge of the vertebral body, then positioned anterolateral to the anterior aspect of the vertebral body. These steps were repeated on the opposite side. Needle locations were then confirmed with negative aspiration and injection of contrast agent to ensure appropriate cephalo-caudad spread between T11 and L2 levels. After this, a 3-mL solution of 2% lidocaine and 1:200,000 epinephrine was injected on each side in 3-minute intervals, and motor function, as well as heart rate, were monitored for changes. This was followed by a test block on each side using 10 mL of a 50:50 mixture of 2% lidocaine and 0.5% bupivacaine. After 5 minutes, patients reporting ≥50% relief of abdominal pain symptoms proceeded with CPN by incremental injection with 10 mL of 98% ethanol on each side, with periodic negative aspiration. After the procedure, patients remained in the prone position for 30 minutes before discharge.

**Figure 2.**
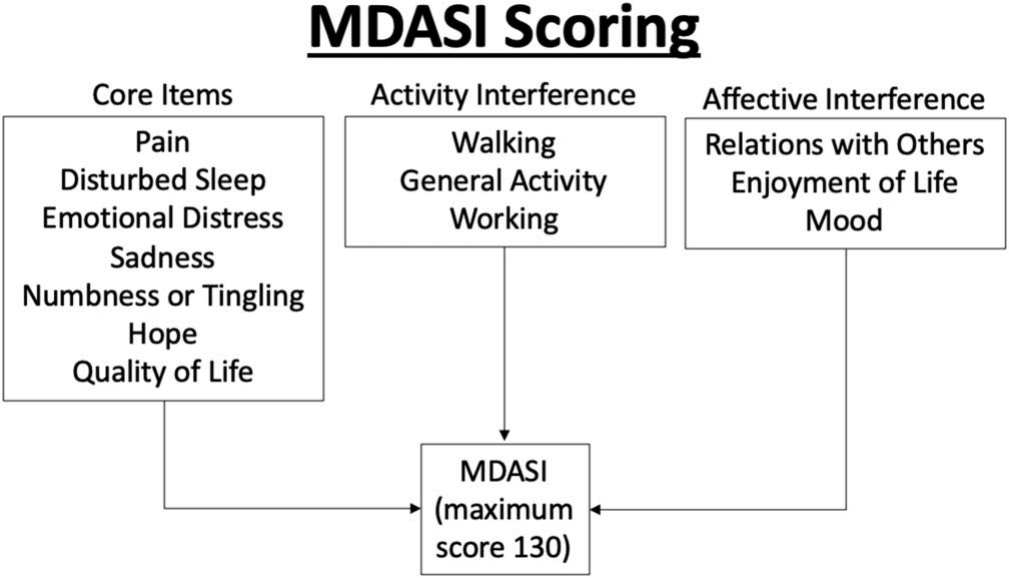
MD Anderson Symptom Inventory Scoring.

All patients completed the MDASI survey before CPN, as well as at each visit after CPN. This survey assessed the patient's symptom severity over a 24-hour period.^3^ Values were recorded on a 0 to 10 numeric rating scale, with 0 constituting “not present,” and 10 constituting “as bad as you can imagine.” A total of 13 symptoms were selected for analysis. These symptoms are categorized as follows: core items (pain, disturbed sleep, emotional distress, sadness, numbness or tingling, hope, and quality of life [QOL]), mean activity interference (general activity, ability to work, and walking ability), and mean affective interference (mood, relations with others, and enjoyment of life) (Fig. 2).

The decision to set the baseline MDASI cutoff at ≤ 45 days before CPN was based on the elective nature of the procedure and the assumption that patients accessing care at this cancer hospital were unlikely to reside nearby. Using common clinical follow-up periods, the 80 days after CPN were demarcated as either 2-week (14 days ± 5 days), 1-month (30 days ± 10 days), or 2-month values (60 days ± 20 days). Each patient who met inclusion criteria had at least 1 follow-up MDASI survey from one of the 3 follow-up periods. Not all patients had 3 consecutive follow-ups within 80 days after CPN, so each of the 3 follow-up periods were studied on an individual basis to evaluate changes in relation to the time that had elapsed since CPN.

Data were analyzed with SAS 9.4 (Cary, NC). The normality of the data was assessed visually with histograms as well as the Shapiro–Wilks Test. Significance of the primary outcome, the difference between post- CPN and pre-CPN MDASI scores, was assessed based on both the paired *t* test as well as its nonparametric equivalent, the signed-rank test, unless the data were not normally distributed, in which case only the signed-rank test was used. A 2-sided *P*-value of ≤0.05 was considered statistically significant. The minimal clinically important difference (MCID) was set at 1-point change based on a range of 0.98 to 1.21 found in previous studies.^3,8,13^

To reduce the risk of multiple testing false positives, the data were analyzed first for the overall MDASI score for all cancers at each time point. When significant, this was followed by subset analysis by the symptom inventory section followed by subsection. A subset analysis was also conducted by individual cancers and grouped cancer stages with sample sizes greater than 8, again starting with the overall MDASI score, and moving to subset analysis if results were significant. The analysis was assessed by the change in MDASI score post-CPN minus pre-CPN. Further analysis was performed for each of the 13 symptoms included in the MDASI as well as the core, affective, and activity grouped interference categories on the MDASI.

Significance of days to CPN or death by the cancer group was assessed by the Kruskal–Wallis Test, the nonparametric equivalent to analysis of variance. The post-hoc analysis for the difference between groups of significant results was assessed by the Dwass, Steel, Critchlow–Fligner Method. Death by the cancer type was assessed by the χ^2^ test. A comparison of the change in the MDASI score from baseline was analyzed with linear regression. Improvement in the MDASI score—a change of less than 0—was analyzed with binary logistic regression.

## 3. Results

The baseline characteristics of the 173 patients who met inclusion criteria are listed in Table 1. A total of 82 patients had primary pancreatic cancers, whereas 91 had nonpancreatic primaries that included 14 hepatobiliary cancer patients and 43 nonpancreatic, nonhepatobiliary (NPNH)-gastrointestinal cancer patients. The remaining 34 patients had various other primary malignancies or unknown primary cancers. Within the 82 pancreatic cancer patients, 9 had stage I or II malignancies, 50 had stage III or IV malignancies, and the remaining 23 had unknown staging.

**Table 1:** Patient characteristics by cancer type.

Variable, Mean (SD)	Cancer stage	n	Years CPN to death	Years diagnosis to CPN	Years diagnosis to death	Deaths, n (%)
All	All	173	0.98 (0.93)	1.56 (2.70)	2.53 (2.88)	110 (64%)
Pancreatic	All	82	0.99 (0.88)	0.77 (1.26)	1.75 (1.52)	55 (67%)
	I & II	9	1.02 (1.05)	1.25 (1.11)	2.27 (1.70)	5 (56%)
	III & IV	50	1.01 (0.91)	0.62 (0.84)	1.60 (1.17)	35 (70%)
	Unknown	23	0.95 (0.78)	0.90 (1.91)	1.85 (2.05)	15 (65%)
Nonpancreatic, nonhepatobiliary gastrointestinal	All	43	0.98 (0.99)	1.70 (1.91)	2.68 (2.35)	28 (65%)
Hepatobiliary	All	14	0.73 (0.82)	0.50 (0.58)	1.22 (1.04)	9 (64%)
Others/unknown*	All	34	1.06 (1.03)	3.71 (4.73)	4.78 (4.77)	18 (53%)
*P*-value		NA	0.58†	<0.0001†‡	<0.0001†‡	0.54§

* Kidney n = 3, prostate n = 2, endometrial n = 2, ovarian n = 1, adrenal n = 1, unknown n = 10.

† Kruskal–Wallis Test.

‡ Dwass, Steel, Critchlow–Fligner Method: (pancreatic and hepatobiliary) < (GI and others/unknown).

§ χ^2^ test.

CPN, celiac plexus neurolysis.

**Figure 3.**
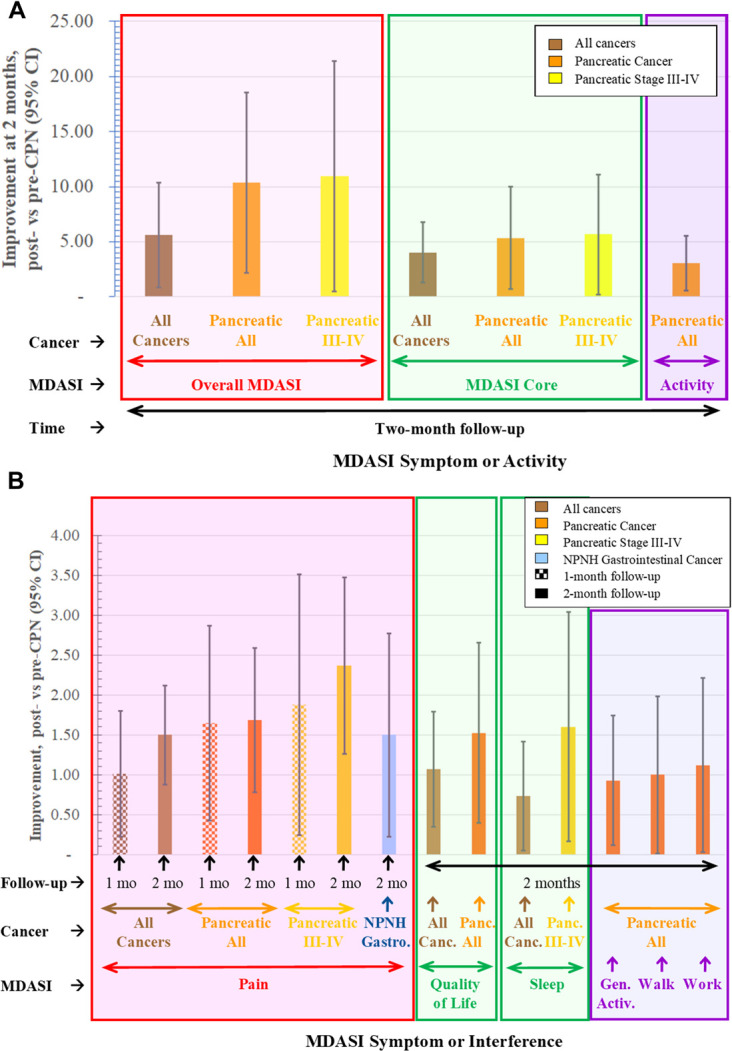
Statistically significant improvements in the MDASI score by the cancer type with the 95% confidence interval. The negative difference (postminus pre-CPN) was inverted for ease of reading. Figure 3A shows improvements in the overall MDASI score, along with the 3 main sections: core, activity interference, and affective interference (not significant). These were significant at 2 months, but not 1 month or 2 weeks. Figure 3B shows the subsections of core and activity interference that were significant. It also includes improvements to pain at different levels and cancer types, although the overall MDASI score was not significant for some of them. Because the same subsection appeared significant across multiple time points and cancer types, it was determined that this could not have occurred by random chance. CPN, celiac plexus neurolysis; MDASI, MD Anderson Symptom Inventory

Statistically significant changes for the entire data set are shown in Figure 3 and Tables 1 and 2. There was no significant difference by the cancer type in the percent of death or in the years from CPN to death. Pancreatic and hepatobiliary cancers had significantly shorter years from diagnosis to CPN and from diagnosis to death compared with NPNH-gastrointestinal and other/unknown cancers (*P* < 0.0001 for each). At the endpoint of our study, 59% of all patients saw an improvement, with 48% having an overall improvement of more than 5 points and 42% having an improvement of 10 points or more. The data set showed a 5.88 point decrease in the total MDASI score from baseline to 2-month follow-up (95% confidence interval −11.1 to −0.68; *P* = 0.028). Pain decreased by 1.01 points from baseline to 1-month follow-up (−1.80 to −0.22; *P* = 0.010), a median change of 9%, and by 1.50 points from baseline to 2-month follow-up (−2.12 to −0.88; *P* < 0.0001), a median change of 14%. Sleep improved by 0.73 points from baseline to 2-month follow-up (−1.42 to −0.04; *P* = 0.044). Quality of life improved by 1.07 points from baseline to 2-month follow-up (−1.80 to −0.35; *P* = 0.0035). All other individual categories of the MDASI were not found to have any statistically significant improvement or worsening at any point after CPN when evaluating the entire data set.

**Table 2:** Mean change in MDASI by the cancer type.

Mean change from baseline (SD)	All Cancers, N = 173			All pancreatic (including 23 patients with unknown staging) n = 82			Pancreatic stages I & II, n = 9			Pancreatic stages III & IV, n = 50			Gastrointestinal, n = 43			Hepatobiliary, n = 14		
	2 week, n = 36*	1 month, n = 77	2 month, n = 112	2 week, n = 20	1 month, n = 34	2 month, n = 57	2 week, n = 3	1 month, n = 2	2 month, n = 6	2 week, n = 11	1 month, n = 24	2 month, n = 35	2 week, n = 9	1 month, n = 22	2 month, n = 22	2 week, n = 2	1 month, n = 4	2 month, n = 9
Baseline MDASI	55.4 (26.3)			52.5 (26.5)			47.3 (19.0)			51.8 (27.5)			60.7 (27.1)			68.9 (22.7)		
Change in MDASI	−4.3 (26.5)	−0.5 (30.1)	**−5.9 (27.5)** †	−4.0 (28.1)	−3.4 (33.6)	**−10.4 (31.0)**	28 (33.9)	2.0 (11.3)	31.9 (7.2)	−13.1 (21.3)	−5.5 (34.3)	**−11.0 (30.4)**	0.7 (29.3)	−0.5 (20.9)	−4.8 (21.0)	−1.0 (2.8)	3.5 (40.5)	−5.6 (22.2)
Activity interference	0.0 (8.5)	1.4 (10.4)	−1.0 (8.9)	−0.1 (9.1)	0.1 (11.0)	**−3.1 (9.5)**	7.7 (9.1)	5.5 (4.9)	−0.3 (8.1)	−2.6 (7.7)	−1.4 (10.7)	−3.1 (10.4)	0.4 (6.4)	2.7 (7.3)	0.0 (6.6)	7.5 (4.9)	0.3 (19.2)	0.0 (10.9)
General activity	0.4 (3.0)	0.4 (3.7)	−0.3 (2.9)	0.5 (3.3)	0.1 (4.0)	**−0.9 (3.1)**	2.7 (2.9)	2.5 (2.1)	−0.2 (3.1)	−0.8 (2.9)	−0.5 (3.9)	−1.0 (3.4)	0.0 (2.6)	0.5 (2.7)	0.3 (2.5)	2.5 (0.7)	0.0 (7.0)	−0.3 (3.4)
Walking	−0.3 (3.2)	0.7 (3.8)	−0.3 (3.6)	−0.3 (3.0)	−0.2 (3.8)	**−1.0 (3.7)**	1.0 (1.0)	1.5 (0.7)	−0.3 (3.4)	−1.3 (3.0)	−0.6 (3.6)	−0.8 (4.0)	0.0 (2.4)	1.1 (2.9)	−0.7 (2.9)	1.5 (3.5)	0.0 (7.0)	−0.3 (4.4)
Working	0.0 (4.6)	0.3 (4.4)	−0.4 (3.9)	−0.2 (5.1)	0.2 (4.3)	**−1.1 (4.1)**	4.0 (5.3)	1.5 (2.1)	0.2 (3.4)	−0.5 (4.4)	−0.3 (4.2)	−1.3 (4.4)	0.4 (3.8)	1.1 (3.8)	0.5 (3.3)	3.5 (0.7)	0.3 (7.3)	0.7 (5.1)
Affective interference	0.2 (8.3)	−0.2 (9.0)	−0.8 (8.7)	0.9 (8.5)	−0.4 (9.9)	−2.0 (9.0)	10.6 (12.9)	0 (4.2)	−1.5 (6.5)	−1.9 (6.9)	−0.1 (10.3	−2.2 (9.7)	−1.3 (10.2)	−1.0 (7.6)	−0.6 (7.8)	−0.5 (4.9)	−1.5 (8.9)	−0.8 (8.3)
Enjoyment of life	−0.1 (3.2)	0.0 (3.5)	−0.6 (4.2)	0.1 (3.6)	−0.4 (3.6)	−0.9 (4.5)	3.0 (4.0)	0.5 (2.1)	1.7 (2.8)	−0.9 (3.2)	0.0 (3.5)	−0.9 (4.9)	−0.9 (3.4)	0.4 (2.4)	−0.7 (3.5)	2.0 (1.4)	−0.5 (7.5)	−1.3 (4.0)
Mood	0.1 (3.1)	−0.1 (3.5)	−0.3 (3.1)	0.1 (3.0)	0.0 (3.7)	−0.6 (3.3)	4.0 (5.3)	−0.5 (0.7)	1.2 (3.8)	−0.5 (1.6)	−0.1 (3.6)	−1.0 (3.4)	0.6 (3.9)	−1.1 (2.9)	−0.1 (2.8)	−1.0 (2.8)	2.0 (3.2)	−0.8 (3.5)
Relations with others	0.2 (3.4)	−0.1 (3.8)	0.1 (3.3)	0.8 (3.3)	0.0 (3.7)	−0.5 (3.5)	3.7 (3.8)	0.0 (1.4)	−1.3 (2.9)	−0.5 (3.1)	0.1 (3.8)	−0.3 (3.7)	−1.0 (4.1)	−0.2 (3.8)	0.2 (2.6)	−1.5 (3.5)	−3.0 (5.7)	1.3 (2.0)
Core	−4.5 (13.5)	−1.8 (15.7)	**−4.0 (14.7)**	−4.8 (14.0)	−3.0 (16.8)	**−5.4 (17.5)**	9.7 (11.9)	−3.5 (2.1)	6.0 (18.7)	−8.6 (12.6)	−4.0 (17.5)	**−5.7 (15.8)**	1.5 (14.0)	−2.3 (12.3)	−4.2 (9.9)	−8.0 (2.8)	4.8 (18.4)	−4.8 (6.8)
Emotional distress	−0.8 (3.5)	−0.4 (3.7)	−0.5 (3.3)	−0.9 (4.1)	−0.5 (3.7)	−0.6 (3.7)	3.0 (6.1)	0.5 (0.7)	3.2 (3.9)	−1.4 (3.7)	−0.7 (3.6)	−1.0 (3.0)	0.4 (2.9)	−0.5 (3.7)	−0.8 (2.6)	−3.5 (0.7)	−1.0 (3.9)	−1.2 (3.6)
Hope	−0.4 (3.5)	0.1 (3.7)	−0.5 (3.7)	−0.2 (3.6)	0.6 (3.9)	−0.5 (3.9)	1.0 (1.7)	0.5 (0.7)	−0.3 (3.2)	−1.1 (3.8)	0.0 (3.8)	0.1 (3.8)	−0.3 (3.8)	0.0 (3.1)	−0.5 (3.6)	−2.0 (4.2)	0.5 (4.1)	−1.1 (3.5)
Numbness/Tingling	−1.1 (3.2)	−0.1 (3.2)	0.4 (3.4)	−0.7 (2.9)	−0.1 (3.3)	0.1 (3.7)	−0.7 (2.5)	−4.5 (3.5)	−2.3 (3.4)	−1.4 (3.3)	0.4 (3.0)	0.4 (3.6)	−0.4 (4.4)	−0.5 (3.1)	0.3 (3.4)	−4.0 (0.0)	−1.5 (1.9)	0.0 (2.0)
Pain	−0.4 (3.3)	**−1.0 (3.5)**	**−1.5 (3.3)**	−0.5 (3.0)	**−1.6 (3.5)**	**−1.7 (3.4)**	2.0 (3.6)	0.0 (1.4)	1.5 (4.5)	−0.2 (2.7)	**−1.9 (3.9)**	**−2.4 (3.2)**	1.0 (2.3)	−0.5 (3.6)	**−1.5 (2.9)**	1.5 (4.9)	−0.5 (1.7)	−0.7 (2.3)
Quality of life	−0.4 (3.9)	0.2 (4.1)	**−1.1 (3.8)**	−0.2 (3.4)	0.4 (4.7)	**−1.5 (4.3)**	3.3 (1.5)	0.0 (1.4)	−1.2 (3.9)	−0.9 (3.8)	0.4 (4.4)	−0.8 (4.0)	−0.1 (5.1)	0.0 (3.1)	−0.7 (2.6)	2.0 (1.4)	3.3 (5.0)	−1.3 (2.2)
Sadness	−0.8 (3.4)	−0.4 (3.6)	−0.1 (3.0)	−1.4 (3.6)	−0.8 (3.6)	−0.1 (3.5)	1.0 (1.7)	0.0 (0.0)	2.7 (4.2)	−1.6 (3.7)	−0.9 (3.6)	−0.4 (2.9)	−0.1 (4.0)	−0.9 (3.7)	−0.2 (2.6)	2.5 (0.7)	3.0 (4.8)	−0.7 (2.4)
Disturbed sleep	−0.7 (3.1)	−0.3 (3.5)	**−0.7 (3.7)**	−1.2 (2.9)	−1.0 (3.3)	−1.0 (4.2)	0.0 (3.6)	0.0 (0.0)	2.5 (4.2)	−2.0 (2.0)	−1.3 (3.8)	**−1.6 (4.2)**	1.1 (3.0)	0.2 (3.4)	−0.7 (2.9)	−4.5 (3.5)	1.0 (5.2)	0.2 (1.6)

* Several patients have data at multiple time points, so counts do not sum up to total.

† Bold values are statistically significant.

MDASI, MD Anderson Symptom Inventory.

Looking only at pancreatic cancer patients, the overall MDASI score decreased by 10.4 points from baseline to 2-month follow-up (−18.6 to −2.16; *P* = 0.012). Although the change from baseline to 2-week follow-up for this group decreased by 4.28 points (−16.6 to 8.56; *P* = 0.46) and by 3.38 points from baseline to 1-month follow-up (−14.9 to 8.16; *P* = 0.45), neither of these changes were found to be statistically significant. The category grouping for activity interference in the MDASI, which is comprised of walking, general activity, and working, improved by 3.05 points from baseline to 2-month follow-up for all pancreatic cancer patients (−5.57 to −0.54; *P* = 0.011).

Individual element analysis for pancreatic cancer patients showed improvements in numerous categories. Pain decreased by 1.65 points from baseline to 1-month follow-up (−2.85 to −0.44; *P* = 0.010) and 1.68 points from baseline to 2-month follow-up (−2.59 to −0.78; *P* < 0.001). The QOL improved by 1.53 points from baseline to 2-month follow-up (−2.65 to −0.40; *P* = 0.0064). Walking improved by 1.00 points from baseline to 2-month follow-up (−1.99 to −0.01; *P* = 0.040). General activity improved by 0.93 points from baseline to 2-month follow-up (−1.74 to −0.12; *P* = 0.017). The ability to work was the final category to show statistically significant changes for pancreatic cancer patients, improving by 1.12 points from baseline to 2-month follow-up (−2.21 to −0.03; *P* = 0.034).

For pancreatic cancer patients with Stage I or II tumors, the MDASI was not found to be significant for any of the post-CPN survey periods. There were limited numbers of patients who had data to compare baseline with 2-week follow-up (n = 3), baseline with 1-month follow-up (n = 2), and baseline with 2-month follow-up (n = 6).

There were 3 categories on the MDASI that showed statistically significant changes in pancreatic cancer patients with stages III or IV. From baseline to 1-month follow-up, pain decreased by 1.88 points (−3*.*46 to −0.29; *P* = 0.024). From baseline to 2-month follow-up, pain decreased by 2.37 points (−3.46 to −1.28; *P* < 0.0001), whereas sleep improved by 1.60 points (−3.02 to −0.18; *P* = 0.025).

Changes among hepatobiliary cancer patients were not found to be statistically significant in any category, at any point after CPN. There were limited numbers of patients who had data to compare baseline to 2-week follow-up (n = 2), baseline to 1-month follow-up (n = 4), and baseline to 2-month follow-up (n = 9).

Among NPNH-gastrointestinal cancer patients' responses, pain improved by 1.50 points (−2.73 to −0.27; *P* = 0.018) from baseline to 2-month follow-up. Other changes on the MDASI were not found to be statistically significant.

**Figure 4.**
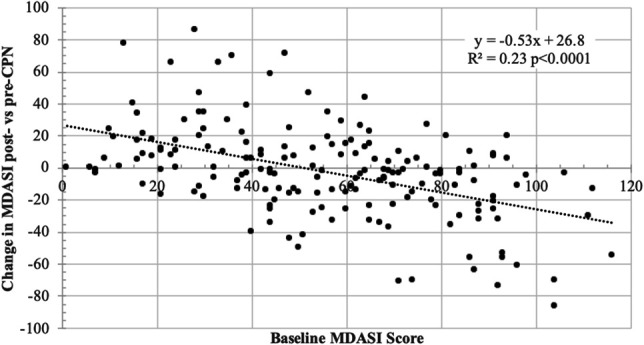
Linear regression of baseline MDASI vs change in MDASI shows that patients with higher baseline MDASI scores show a greater change in MDASI. Similarly, binary logistic regression shows that for every 10-point increase in baseline MDASI, the odds of showing an improvement (ie, a change of less than 0) were 1.44 (95% CI 1.25–1.65). MDASI, MD Anderson Symptom Inventory; CI, confidence interval.

Comparing baseline MDASI with change in MDASI using linear regression showed that patients with higher starting MDASI scores had greater improvement than patients with lower baseline MDASI scores (Fig. 4). Similarly, dichotomizing the change in the MDASI score to “showing an improvement” (ie, a negative change) vs not, a binary logistic regression showed an odds ratio of 1.44 (95% confidence interval 1.25–1.65) for every 10-point increase on the MDASI.

## 4. Discussion

The celiac plexus is a rich collection of nerve fibers located anterolateral to the aorta, between the T12-L2 vertebral bodies.^12^ In total, the plexus communicates with the gastrointestinal tract, beginning at the distal esophagus and continuing through the mid-transverse colon, including the pancreas, liver, biliary tract, spleen, and kidneys.^15^ Neurolytic disruption of these signaling pathways using CPN has focused on mitigating pancreatic cancer pain for over a century, but CPN's utility beyond pancreatic cancer pain has not been well established. In this retrospective study of 173 unresectable abdominal malignancy patients who received CPN, the symptoms found to improve were pain, sleep, QOL, general activity, walking, and working. These changes occurred as early as 2 weeks after CPN, but most were not significant until 2 months after the procedure. The lack of significant change in the overall MDASI score at 2 weeks or 1 month but significant change in the overall MDASI score at 2 months suggests that improvements are not prominent until this time. Among the pancreatic cancer patients in our study, those with late-stage tumors were more likely to experience significant improvement. Among nonpancreatic cancer patients, those with NPNH-gastrointestinal cancer were found to benefit from CPN. There were 14 hepatobiliary cancer patients, 9 early-stage pancreatic cancer patients, and 34 patients with other cancers who met inclusion criteria for the study but were not found to have any significant changes likely because of limitation in sample sizes.

The primary indication for CPN remains abdominal pain. An abundance of the available literature shows that CPN is efficacious in reducing pain, but there are wide variances in the amount of time it takes for patients to feel meaningful relief. Some studies state that 88% of patients experience a reduction in pain after just 24 hours after CPN.^24^ Others find that it takes 2 weeks for 89% of patients to have adequate pain relief.^6^ Longitudinal studies extending to 3 months post-CPN reveal that this is when 90% of patients report partial, if not complete, relief from pain^9^. Not every patient should expect such promising results, however. Safer estimates show that the average patient may have a 35% decrease in pain scores after 1 month.^24^ In our analysis, the median change in pain at 1-month follow-up amounted to a 9% improvement and to a 14% improvement at 2 months. The 1-month follow-up after CPN was the earliest that a statistically significant change was found. This was specific for all cancers overall, all pancreatic cancer patients, and even more specific for late-stage pancreatic cancer patients. Two months after CPN, late-stage pancreatic cancer patients continued to have significant pain relief, whereas NPNH-gastrointestinal cancer patients experienced their first period of significant pain relief. Based on our findings, we support the conservative timeframe of 1 month being when pain is first noticeably reduced and believe that the change in pain after CPN remains reduced for at least another month.

A previous study found sleep to improve as early as 2 weeks after CPN.^18^ In our study, we followed sleep past 2 weeks and found that it improves at 2-month follow-up in late-stage pancreatic cancer patients and all cancers overall. This is a finding that we are not aware of in other studies. Poor sleep has been linked to increased risk of morbidity and mortality, as well as decreased QOL in cancer patients.^9,23^ Although it can be difficult to tease apart whether a patient's sleep disturbance is due to pain, or a result of other factors, CPN should be considered to prevent worsening of conditions for patients with sleep issues.^9^

Another MDASI item that changed significantly after CPN was QOL. This differs from a previously conducted randomized control trial comparing QOL between CPN patients and opioid-exclusive patients, concluding that there is no difference in QOL at 2-months.^22^ That study included a total of 36 pancreatic cancer patients, whereas our study included a total of 112 patients, 57 of whom had pancreatic cancer. Studying a larger number of patients uncovered that CPN likely improves QOL in pancreatic cancer patients, but it is important to note the timing of the change. It took 2 months for a significant improvement in QOL to be discovered, coinciding with an overall improvement in pain and sleep. Interestingly, these 3 items (QOL, pain and sleep) are closely correlated, making it difficult to distinguish which item is the chief influencer among the 3 in a given patient.^14^

A major category to change after CPN was the activity interference grouping on the MDASI comprised general activity, walking, and working, all of which changed at some point during the study. Other studies have found fluctuations in functional changes making it difficult to determine if and when a particular patient may experience improvement.^1,4,18^ In our study, the pancreatic group experienced their first bout of improved general activity at 2 months, along with improvements in walking and working. Based on these findings, it is likely that it will take pancreatic cancer patients 2 months to demonstrate functional improvements after CPN, but the same cannot be said for patients with other cancers.

The data set made clear that patients could expect to receive the most variety of benefits from CPN at the 2-month follow-up. Not only did total MDASI scores significantly improve during this period, but at least 3 of 13 individual elements in the MDASI also improved. Statistical significance was not found overall or for most individual symptoms at 2 weeks or 1 month. It is unclear if the failure to find a significant difference during these time periods was due to the absence of a difference or due to low statistical power from the limited sample sizes for these time periods. Larger future studies focused on these shorter periods could help to clarify this. A key distinction to be made based on our findings is that, although the overall improvements up to 1-month follow-up were not significant, the benefits of CPN overlap with one another as time goes on, compounding to result in a favorable scenario for patients by the end of our study.

As with any study of CPN, pain emerged as one of the leading symptoms to improve at numerous endpoints in the study. Other studies have aimed to evaluate pain by tracking changes in opioid consumption, but the retrospective nature of this study made it difficult to establish an opioid-only control group or track individual patients' opioid consumption changes. This limits interpretation of results to patients who are concomitantly treated with traditional opioid medication management. It is important to understand that, for cancer patients experiencing pain, opioids are often considered the first line of defense.^21^ Most patients prescribed opioids before CPN fluctuate in their use of opioids after the procedure, and it is very rare that cancer patients discontinue opioids entirely from their treatment plan, regardless of the type of intervention they receive.^11^ Furthermore, because of the endless variations of opioid formulations in the market, it is difficult to accurately quantify the total dose of opioids administered to a patient within a given day, especially if the patient is self-administering their medications without direct supervision. Widely available morphine equivalent dose calculators have been created in an attempt to standardize measurements of opioid consumption, but recent studies have shown that these calculators are not uniform and can be unreliable.^17^ The formulae used by these calculators frequently neglect opioid tolerance, drug–drug interactions, metabolism of the drugs, and the use of breakthrough pain medications. These overlooked features of chronic opioid use are ubiquitous among patients prescribed opioids for cancer pain and largely affect the interpretation of opioid consumption. Because of these complex factors, we chose instead to use the MDASI questionnaire as a reliable benchmark to follow changes in symptoms to determine CPN's efficacy.

The MDASI questionnaire has been validated in multiple studies, for multiple cancer types, and can be easily administered in multiple languages.^3^ The simplicity of the questionnaire allows it to be self-administered in less than 5 minutes, allowing even patients with high levels of symptom burden to complete the survey without much disturbance. Some limitations of the MDASI are that a standard MCID does not exist, and certain symptoms on the questionnaire may require additional information from the patient. Previous articles using MDASI had MCIDs ranging from 0.98 to 1.21, so we decided to set MCID to a change of 1 point.^3,8,13^ Having an established MCID for MDASI would be beneficial, which is why we recommend that future studies of the symptoms found to be statistically significant in this study be conducted in a prospective manner using an opioid-only control group and using symptom-specific tools with established MCIDs to show both statistical and clinical significance.

One other limitation of this study was that low numbers of patients met inclusion criteria for the for NPNH-gastrointestinal, hepatobiliary, or pancreatic cancer stages I or II. These 3 groups did not demonstrate any statistically significant changes in their overall MDASI score at any time period. However, there are insufficient data to determine if this was due to lack of difference or because of low statistical power from the relatively low sample sizes of n = 9 to 22, n = 2 to 9, and n = 2 to 6, respectively. Therefore, the absence of statistical significance should not be viewed as evidence of ineffectiveness in these groups. With hepatobiliary cancer cases now found to have the fastest-growing cancer death rate in the United States, it is even more imperative to understand if interventional pain therapies such as CPN can be used to reduce symptomatology in this group.^7,16^ Similarly, with the dismal prognosis of even early-stage pancreatic cancers, the potential benefit of CPN seen in late-stage pancreatic cancers may still be relevant for early-stage pancreatic cancers.^4,5,15,18,20,24^ Because of the anatomy of the celiac plexus, we believe that hepatobiliary, early-stage pancreatic, and NPNH-gastrointestinal cancer patients all have an opportunity to benefit from CPN and hope that future studies are conducted with larger populations of these patients.

## Disclosures

Dr. Vinita Singh is a consultant for Releviate LLC. The remaining authors have no conflicts of interest to declare.

Dr. V. Singh is a KL2 scholar at the Georgia, Clinical, and Translational Science Alliance and would like to acknowledge that this article was supported by the National Center for Advancing Translational Sciences of the National Institutes of Health under Award number ULTR002378 and KL2TR002381. The content is solely the responsibility of the authors and does not necessarily represent the official view of the National Institutes of Health.
